# Analysis of the Codon Usage Bias Pattern in the Chloroplast Genomes of *Chloranthus* Species (Chloranthaceae)

**DOI:** 10.3390/genes16020186

**Published:** 2025-02-02

**Authors:** Jisi Zhang, Miao Feng

**Affiliations:** Liaoning Key Laboratory of Development and Utilization for Natural Products Active Molecules, Anshan Normal University, Anshan 114000, China; fengmiao@asnc.edu.cn

**Keywords:** *Chloranthus*, chloroplast genome, codon usage bias, phylogeny

## Abstract

Background: The codon preference of chloroplast genomes not only reflects mutation patterns during the evolutionary processes of species but also significantly affects the efficiency of gene expression. This characteristic holds significant scientific importance in the application of chloroplast genetic engineering and the genetic improvement of species. *Chloranthus*, an ancestral angiosperm with significant economic, medicinal, and ornamental value, belongs to the basal angiosperms. However, the codon usage patterns among *Chloranthus* species have remained unclear. Methods: To investigate codon usage bias and its influencing factors in *Chloranthus* chloroplast genomes, we utilized CodonW, CUSP, and SPSS software to analyze the chloroplast genomes of seven *Chloranthus* species. Results: In this study, we reported and characterized the complete chloroplast genome of the Chinese endemic species *Chloranthus angustifolius*. The phylogenetic tree based on the whole chloroplast genomes showed that *C. angustifolius* is sister to *Chloranthus fortunei*, and the genus *Chloranthus* is divided into two major clades, consistent with previous studies. Our results revealed that the GC content at different codon positions across all seven *Chloranthus* species was less than 50%, with GC1 > GC2 > GC3. Additionally, the average effective number of codons (ENC) values exceeded 45. A total of 10 shared optimal codons were identified, nine of which end with A or U. PR2-plot, ENC-plot, and neutrality plot analyses indicated that natural selection primarily influenced codon usage bias in the chloroplast genomes of *Chloranthus*. Conclusions: We newly obtained the chloroplast genome of *C. angustifolius* and proposed that natural selection played a key role in codon usage patterns in *Chloranthus* species. These findings contribute to our understanding of evolutionary history and genetic diversity within this genus.

## 1. Introduction

Codons play a crucial role in the transmission of genetic information between nucleic acids and proteins in organisms, with most amino acids being encoded by at least two synonymous codons [1]. Codon usage bias (CUB) refers to the preferential and non-random usage of synonymous codons [2]. Influenced by various factors such as mutation, natural selection, genome size, codon position within genes, mRNA folding, protein structure, and tRNA abundance, CUB not only differs among populations within species, families, or kingdoms, but also varies among genes within an organism [3,4]. CUB can impact life processes such as gene transcription and protein translation and folding, reflecting the origin of species or genes and their adaptability to the environment [5]. The analysis of codon usage bias provides valuable insights into the evolutionary processes that shape gene sequences and expression patterns, and helps us to understand how species adapt to their environments and how genes function within organisms [6,7,8].

Chloroplasts function as the primary sites for photosynthesis in green plants and are semi-autonomous organelles endowed with their own genomes and protein synthesis machinery [9]. The chloroplast genome is of moderate size, structurally conserved, and contains a wealth of genetic information. By comparing and analyzing the chloroplast genomes of different species, we can gain insights into the phylogenetic relationships among plant species and explore their evolutionary mechanisms [10,11,12]. Furthermore, compared to nuclear gene transformation, chloroplast gene transformation offers advantages such as high expression efficiency of foreign genes in chloroplasts, site-specific integration, absence of position effects, and genetic stability [13,14]. In the process of protein coding in plant chloroplast genomes, there is a widespread phenomenon of CUB. Previous studies have shown that during long-term evolution, the codon preference of species is influenced by multiple factors such as base mutations and natural selection. These factors jointly determine the pattern of codon usage and further affect the expression efficiency of exogenous genes in chloroplasts [15,16]. Additionally, there are significant differences in codon preference among different species. Therefore, by studying the codon preference of chloroplast genomes, the expression level of exogenous genes in chloroplasts can be optimized, which is of great significance for promoting the application of chloroplast genetic engineering and genetic improvement of species [17,18].

The genus *Chloranthus* (Chloranthaceae), as one of the basal angiosperm lineages, comprises 13 species and 5 varieties, and is distributed from Asian tropical to temperate regions, with a diversity center in China [19,20,21,22,23,24]. The species are perennial herbs or shrubs with high ornamental values, such as *Chloranthus spicatus* (Thunb.) Makino. Eight of them are used as Chinese traditional herbal medicines, with the roots or rhizomes being the main medicinal parts [19]. Recently, secondary metabolites, such as terpenes, amides, sterides, and lignans, have been isolated from the plants of this genus, and modern pharmacological studies have shown that these chemical constituents can be used for anti-tumor, antibacterial, anti-inflammatory, antioxidant, neuroprotection, and other activities [25,26,27,28]. Further, the phylogenetic relationships among this genus have been resolved. For example, Kong et al. (2002) constructed the phylogenetic tree of *Chloranthus* and deem that this genus can be divided into two major clades: one clade containing *Chloranthus erectus*, *C. spicatus*, *Chloranthus serratus*, *Chloranthus henryi*, *Chloranthus sessilifolius*, and *Chloranthus oldhamii*, and the other comprising *C. angustifolius*, *C. fortunei*, *Chloranthus nervosus*, and *Chloranthus japonicus*. [20]. Zhang et al. (2015) employed ITS and four chloroplast regions to support the two clades division [23]. Yao et al. (2023) obtained the new chloroplast genome of *C. nervosus* and also revealed the recognition of the two clades with the highest supporting values [29]. However, there is still a lack of information on the codon usage patterns among *Chloranthus* species.

The aims of this study were to confirm the phylogenetic position of *C. angustifolius* and reveal the codon usage characteristics of the chloroplast genome coding regions in *Chloranthus*. For these purposes, we first sequenced and annotated the complete chloroplast genome of *C. angustifolius*, and then collected chloroplast genomes of six other *Chloranthus* species to reveal the codon usage bias among this genus. Finally, we reconstructed the phylogeny of this genus based on 86 protein-coding genes (CDSs) and the whole chloroplast genome, and compared the topology from relative synonymous codon usage (RSCU) values. This study conducted a systematic and comparative analysis of codon usage characteristics in *Chloranthus* for the first time, which sheds new light in understanding the evolution of this genus and provides a scientific basis for the codon optimization of exogenous genes and the improvement of their expression efficiency in future chloroplast genetic engineering applications within this genus.

## 2. Materials and Methods

### 2.1. Sampling, DNA Extraction, Sequencing, Chloroplast Genome Assembly, and Annotation

The samples were collected from Daozhen County, Guizhou Province, China (28°51′57.45″ N, 107°36′32.27″ E), and cultivated in Anshan Normal University. The voucher specimen (accession no. ZJS_2021086) was deposited in the specimen room of Anshan Normal University (https://www.asnc.edu.cn/, Contact: Ji-Si Zhang, E-mail: zhangjisi@asnc.edu.cn). Total genomic DNA was extracted from silica gel-dried leaves using the modified CTAB method [30] and the Illumina paired-end (PE) library was prepared and sequenced in Nanjing Novogene Biotechnology Co., Ltd., Nanjing, China.

In total, 4 Gb of 150-bp paired-end raw reads were generated and used for chloroplast genome assembly. The quality of raw sequence reads was assessed in FastQC v0.11.9 [31] and the adapters and low-quality reads were filtered using Trimmomatic v0.39 [32]. The clean reads were assembled by GetOrganelle v1.5 [33], and the assembled genomes were checked and visualized in Bandage v0.7.1 [34]. Finally, we obtained the high-quality and complete plastomes. The chloroplast genome of C. angustifolius was sequenced to obtain 13,699,315 raw reads. After filtering, 13,630,820 clean reads were obtained, with an effective rate of 99.5%. The average coverage depth was higher than 1000×, which exceeded the required average sequencing depth of 100× for sequence assembly.

The chloroplast genome of *C. angustifolius* was annotated used GeSeq [35] and Geneious v9.0.5 (http://www.geneious.com/) with *C. japonicus* (NC_026565) as reference. The annotated complete chloroplast genome of *C. angustifolius* was deposited in GenBank (accession number MW581013).

### 2.2. Phylogenetic Analysis

To determine the phylogenetic position of *C. angustifolius*, another six complete chloroplast genomes of *Chloranthus* were downloaded from GenBank, including *C. erectus*, *C. fortunei*, *C. henryi*, *C. japonicus*, *C. nervosus*, and *Chloranthus spicauts*. Seven species (*Sarcandra glabra*, *Cinnamomum camphora*, *Cinnamomum kotoense*, *Liriodendron chinense*, *Piper nigrum*, *Piper longum*, and *Amborella trichopoda*) from basal angiosperm were selected as outgroups according to APG IV [36]. All sequences were aligned with MAFFT v7.409 [37]. The maximum likelihood tree and maximum parsimony were reconstructed using RAxML 8.2.12 [38] and PAUP v.4.0b10 [39], respectively. For the ML analyses, nodal support on the ML tree was evaluated by the rapid BS (1000 replicates). For MP analysis, heuristic searches were conducted with 1000 replicates of random addition, with one tree held at each step during stepwise addition, tree-bisection-reconnection (TBR) branch swapping, MulTrees in effect, and steepest descent off. Bootstrapping was conducted with 1000 replicates with 10 random taxon additions and heuristic search options.

### 2.3. Calculation of Parameters Related to Codon Usage Bias

Sequences with a length greater than 300 bp were selected for subsequent analysis based on annotations, ensuring that the extracted CDS initiated with the start codon ATG and terminated with the stop codons TAA, TAG, or TGA, and that no stop codons appeared prematurely within the sequences. In this study, 51 protein-coding genes were selected and analyzed for the codon usage bias in the *Chloranthus* chloroplast.

Using CodonW1.4.2 software, we calculated parameters such as the effective number of codons (ENC), and relative synonymous codon usage (RSCU) for the protein-coding sequences in the chloroplast genome of *Chloranthus* [40,41]. Additionally, we used the CUSP online software (http://www.bioinformatics.nl/emboss-explorer/, accessed on 1 November 2024) to analyze and tally the A, T, G, and C content at the third position of each codon (A3, T3, G3, C3), as well as the GC content at the first (GC1), second (GC2), and third (GC3) positions of the codons, the overall GC content (GCall), and the G + C content at the third position of synonymous codons (GC3_S_). Pearson correlation was conducted using IBM SPSS Statistics 29.

### 2.4. Neutrality Plot Analysis

Using GC12 (the average of GC1 and GC2) as the ordinate and GC3 as the abscissa, a corresponding scatter plot was drawn. When the slope of the regression curve is close to 1, it indicates that the gene is significantly influenced by mutational pressure; when the slope is close to 0, selection pressure is considered to be the main driving force behind codon usage bias [32,33,34,35,36,37,38,39,40,41,42,43,44].

### 2.5. ENC-Plot Analysis

If the usage frequency of synonymous codons is solely determined by base mutations, then ENC_exp_ = 2 + GC3_S_ + 29/[GC3_S_^2^ + (1 − GC3_S_)^2^], and a standard curve is plotted. Using the GC3 of each gene as the abscissa and the actual ENC value as the ordinate, a scatter plot is drawn for each gene to perform an effective number of codons plotting (ENC-plot) analysis. The scatter plot can be used to determine the causes of codon usage bias [45].

### 2.6. Parity Rule 2 (PR2) Bias Plot Analysis

The PR2-plot is utilized to assess the balance of mutations between the third-position nucleotides G/C and A/T across codons. In this plot, the x-axis represents the ratio of G3/(G3 + C3), while the y-axis represents the ratio of A3/(A3 + T3). The central point (0.5, 0.5) signifies equal frequencies of G = C and A = T, indicating that codon usage bias at this point is predominantly shaped by mutation pressure. Vectors extending from the center to individual data points in the plot illustrate both the direction and magnitude of deviation from mutational equilibrium for each gene [46,47].

### 2.7. Identification of Optimal Codons

The effective number of codons (ENC) values of 51 protein-coding genes in *Chloranthus* were sorted and the top 10% of genes were selected to construct a high-expression gene pool, while selecting the bottom 10% to construct a low-expression gene pool. The relative synonymous codon usage (RSCU) and ΔRSCU values were calculated for both pools. An RSCU greater than 1 indicates that the codon is a high-frequency codon. Codons with a ΔRSCU greater than or equal to 0.08 are defined as high-expression codons. Codons that meet both of these criteria are referred to as optimal codons [48].

## 3. Results

### 3.1. Chloroplast Genome Characters of C. angustifolius

**Figure 1:**
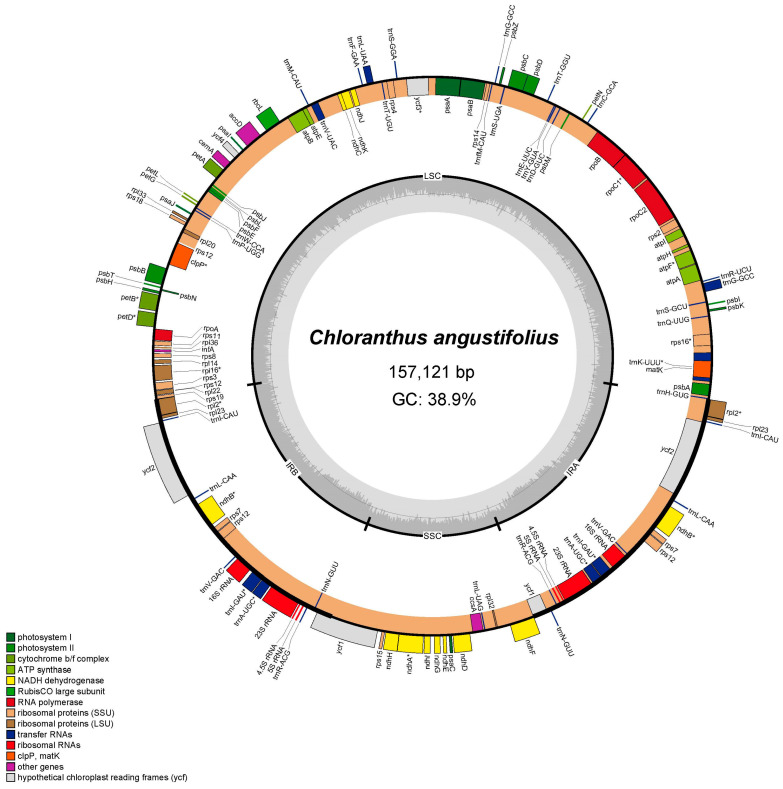
Chloroplast genome map of *C. angustifolius.* Genes located in the outer circle are transcribed in a counterclockwise direction, while those within the inner circle are transcribed clockwise. The dark gray shading in the inner circle indicates regions of high GC content, whereas the light gray shading represents areas with higher AT content.

The complete chloroplast genome of *C. angustifolius* is a typical quadripartite structure and the length is 157,121 bp, with a small single-copy region (SSC) of 18,473 bp, a large single-copy region (LSC) of 86,350 bp, and a pair of inverted repeat regions (IRs) of 26,149 bp (Figure 1). There are 131 genes annotated, including 86 CDSs, 37 transfer RNA (tRNAs) genes, and eight ribosomal RNA (rRNAs) genes. Among the 131 genes, 76 were related to self-replication, including 11 genes related to the large subunit of the ribosome and 14 related to the small subunit of the ribosome. A total of 43 genes were involved in photosynthesis, including 6 related to ATP synthase, 12 to NADH dehydrogenase, 6 to the cytochrome b/f complex, 5 to the PS I system, 14 to the PS II system, and 1 associated with Rubisco. Additionally, 12 genes were annotated as having other (*infA*, *clpP*, *ccsA*, *accD*, *cemA*, and *matK*) or unknown functions (*ycf1*, *ycf2*, *ycf3*, and *ycf4*). Fourteen genes had a single intron (*atpF*, *ndhA*, *ndhB*, *petB*, *petD*, *rpl2*, *rpl16*, *rpoC1*, *rps12*, *trnA*-UGC, *trnI*-GAU, *trnK*-UUU, *trnL*-UAA, and *trnV*-UAC), while *clpP* and *ycf3* contained two introns (Table S1).

### 3.2. Phylogenetic Analysis

**Figure 2:**
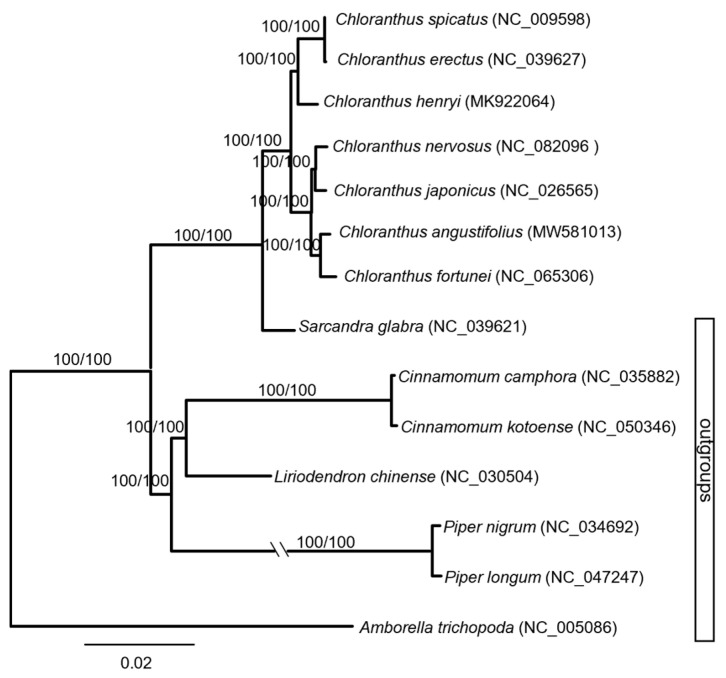
Phylogenetic tree of *Chloranthus* based on the complete chloroplast genome.

Maximum parsimony (MP) and maximum likelihood (ML) trees were constructed based on the whole chloroplast genome (Figure 2). The monophyly of the genus *Chloranthus* had been well supported (MP-BS = 100%, ML-BS = 100%). The seven species sampled here were mainly divided into two major clades with the highest supporting values (MP-BS = 100%, ML-BS = 100%). *C. erectus*, *C. spicatus,* and *C. henryi* were well supported as a clade (MP-BS = 100%, ML-BS = 100%), and *C. erectus* and *C. spicatus* were sisters (MP-BS = 100%, ML-BS = 100%). The other four species formed another clade with the highest supporting values (MP-BS = 100%, ML-BS = 100%). Within this clade, *C. angustifolius* and *C. fortunei* formed a subclade, and *C. nervosus* and *C. japonicus* formed another subclade.

### 3.3. Calculation of Parameters Related to Codon Usage Bias

**Figure 3:**
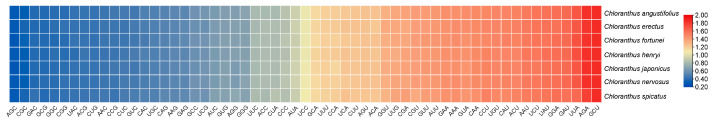
Heat map of codon usage bias in the plastomes of seven *Chloranthus* species under RSCU values.

The codon usage bias of CDSs within *Chloranthus* is shown in Figure 3. There are 30 preferred codons (RSCUS > 1), 2 non-preferred codons (RSCU = 1), and 27 less used codons (RSCU < 1). The preferred codons generally ended in A or U, except for UUG.

The 51 selected protein-coding sequences from the seven *Chloranthus* chloroplasts are presented in Table S2. The GC distribution pattern was generally consistent in all the seven species. The GC content (GCall) of *C. erectus* and *C. henryi* was 39.2%, and the GCall of the other species (*C. angustifolius*, *C. nervosus*, *C. spicatus*, *C. japonicas,* and *C. fortunei*) was 39.3% (Table 1). The GC contents of the first, second, and third positions in the codons were all less than 50%, with the highest at the first position (47.43%), the lowest at the third position (30.07%), and uniformly 40.30% at the second position. The average ENC values of *Chloranthus* ranged from 46.23 to 46.32.

To determine the key factors influencing codon usage bias, the correlation analysis of base composition parameters was conducted for the chloroplast genomes of seven *Chloranthus* species. The results showed that there was no significant correlation between GC1, GC2, and GC3 pairwise (Table 2 and Table S3). Specifically, the effective number of codons (ENC) showed a significant positive correlation with the codons number in all the seven species. Moreover, the ENC had a significant negative correlation with the GC2 content in *C. erectus, C. henryi,* and *C. fortunei*, while having a significant positive correlation with the GC3 content in *C. japonicus, C. erectus,* and *C. henryi*. These results indicated that the base composition similarity is relatively low at different positions of the chloroplast genome in *Chloranthus* species.

### 3.4. Neutrality Plot Analysis

**Figure 4:**
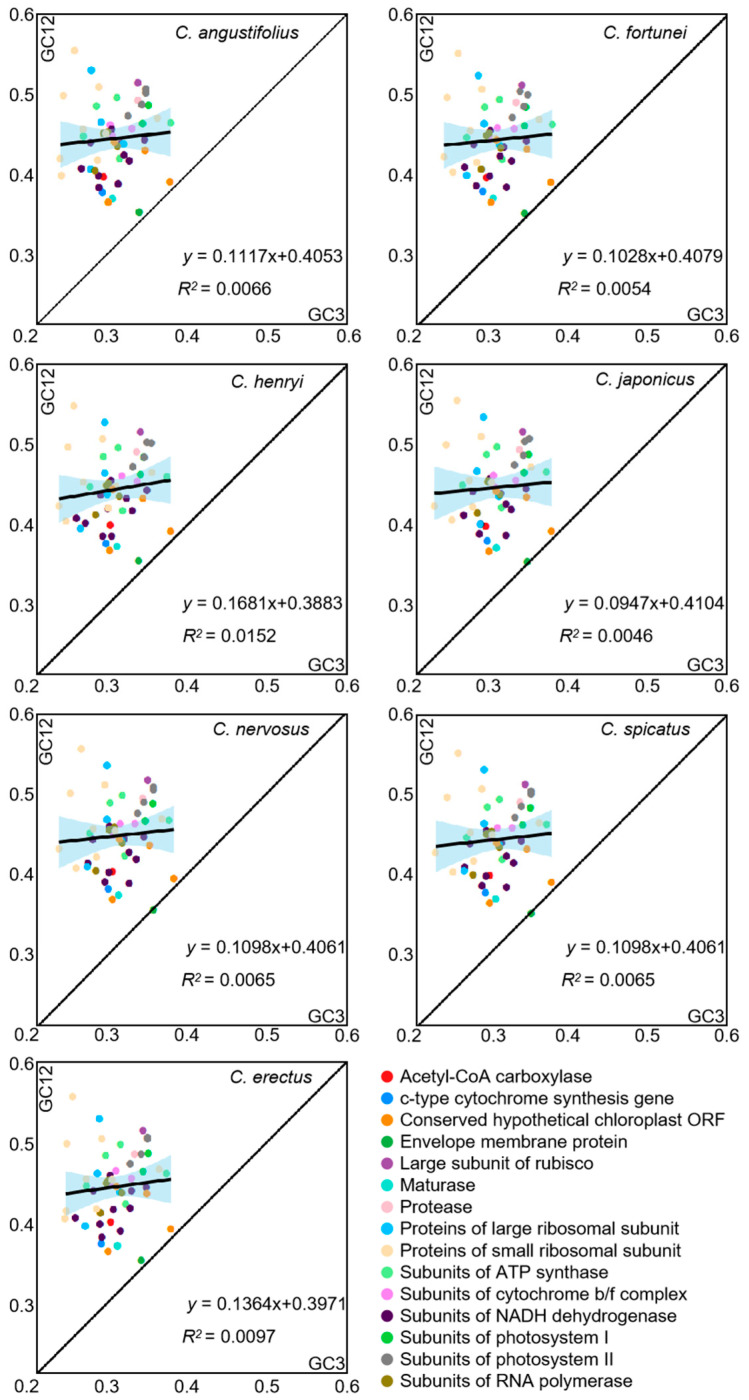
Neutrality plot of chloroplast CDSs in different species. The black line in the plot illustrates the correlation trend, with the corresponding equation displayed at the bottom of the graph.

To reflect the extent to which natural selection and mutational pressure influence codon bias, neutrality plot analysis between GC12 and GC3 was carried out in the chloroplast genome of *Chloranthus* (Figure 4). The majority of genes were distributed above the diagonal, with only a few genes (*cemA* and *ycf2*) lying along the diagonal. The slope ranges from 0.0947 to 0.1681, and *R^2^* ranges from 0.0046 to 0.0152, which indicated significant differences in base content at different positions and a weak correlation between GC12 and GC3. The influence of mutation on codon usage patterns accounts for 0.0947 to 0.1681, while the influence of selective pressure on codon usage patterns accounts for 0.8319 to 0.9053. This result showed that the codon preference in the chloroplast genomes of *Chloranthus* species is primarily influenced by selective pressure.

### 3.5. ENC-Plot Analysis

**Figure 5:**
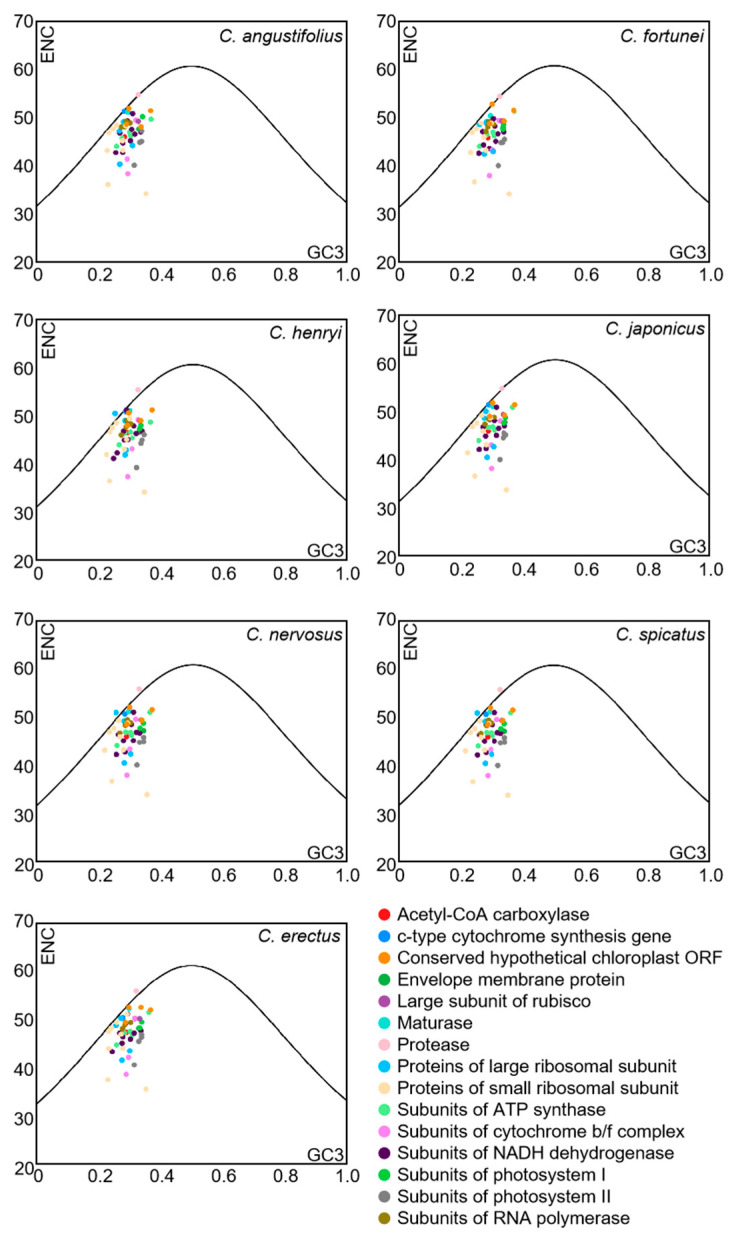
ENC-plot analysis of chloroplast CDSs in *Chloranthus* species. If a data point is significantly distant from the standard curve, this indicates that the codon usage bias of chloroplast coding sequences is predominantly influenced by natural selection.

**Figure 6:**
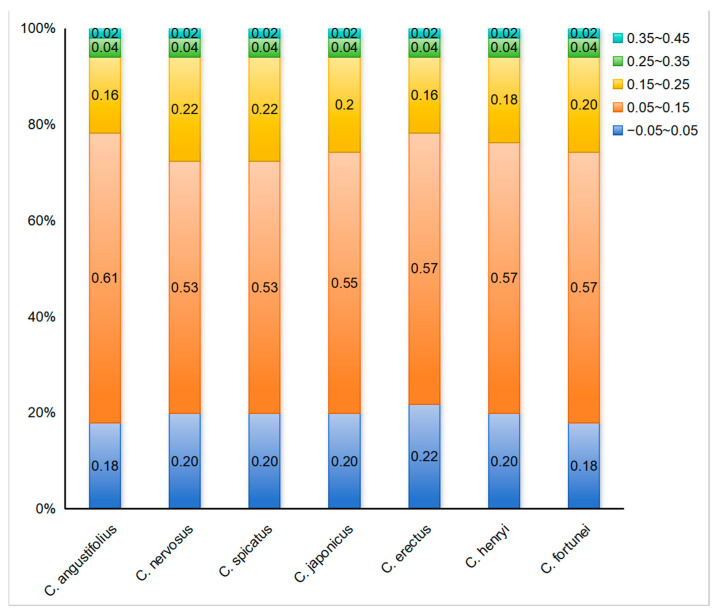
Distribution of ENC ratio frequency in *Chloranthus.* If codon usage bias is predominantly influenced by mutational pressure, then the ENC ratio of most genes should fall within the range of −0.05 to 0.05.

To further reflect the influence of mutation or selective pressure on codon usage preference, the ENC-plot analysis was employed to indicate the magnitude of the difference between ENC_obs_ and ENC_exp_. The ENC-plot graphs of the chloroplast-encoded genes of the seven *Chloranthus* species were similar, with most genes located below the standard curve, indicating a significant deviation between ENC_obs_ and ENC_exp_ for the majority of the genes (Figure 5). Additionally, there were 9–11 genes with ENC ratios distributed between −0.05 and 0.05, accounting for 18%–22% of the 51 selected CDSs (Figure 6), and ENC_obs_ was relatively close to ENC_exp_. There were 40–42 genes with ENC ratios outside the range of −0.05 to 0.05, accounting for 78% to 82% of the 51 selected CDSs. For these genes, there was a significant deviation between ENC_obs_ and ENC_exp_, which indicated that their codon preference was more influenced by natural selection. These results suggested that the codon usage preference in the chloroplast genomes of *Chloranthus* species was less influenced by mutation and more influenced by natural selection.

### 3.6. Parity-Rule 2 (PR2) Bias Plot Analysis

**Figure 7:**
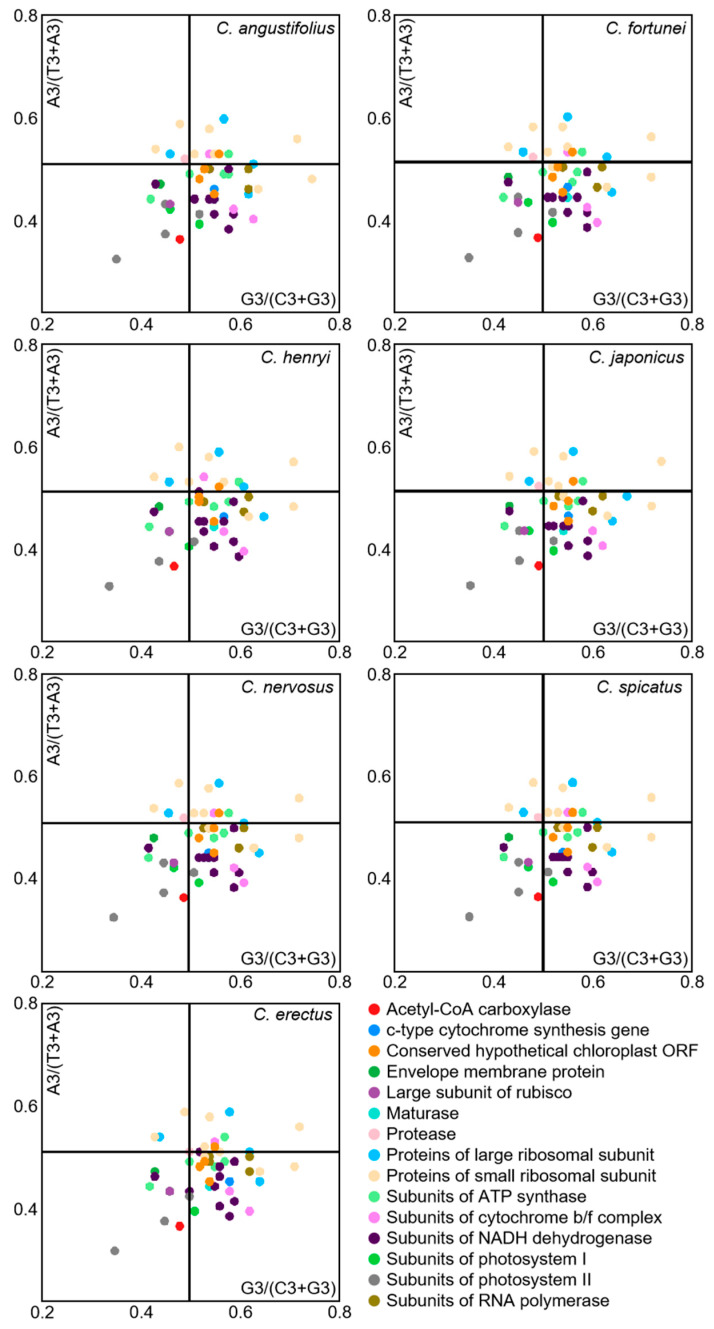
PR2-plot analysis of chloroplast CDSs in *Chloranthus* species. The GC bias is plotted on the *x*-axis, while the AT bias is plotted on the *y*-axis.

Under the sole effect of mutational pressure, the randomness of mutations makes the probability of the third position base in a codon being A/T or C/G equal. However, when influenced by natural selection pressure, the usage frequencies of A/T or G/C become unequal. Codon preference analyses in the chloroplast genome of *Chloranthus* were conducted through PR2-plot graphing (Figure 7). The scatter distribution in the four regions of the PR2-plot were uneven, with most genes located in the lower half of the plot, particularly with the highest number of genes located in the lower right quadrant. This observation indicated that the usage frequency of the third base U is higher than that of A, and the usage frequency of G is higher than that of C. Moreover, the third base of codons in the chloroplast genome of *Chloranthus* exhibits a certain preference in selection, which is influenced by a combination of selective pressure and other factors, with natural selection being the primary factor.

### 3.7. Identification of Optimal Codons

**Figure 8:**
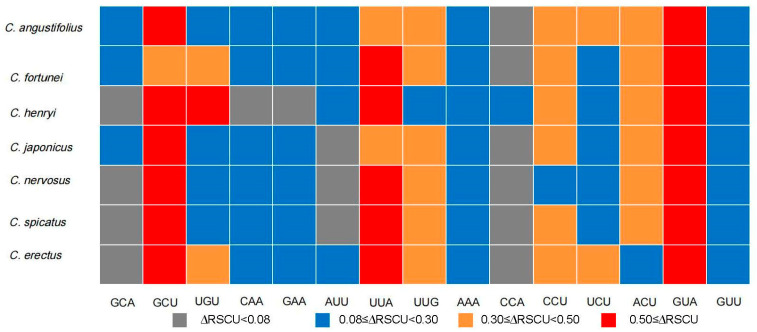
Optimal codons used in the chloroplast genomes of *Chloranthus*.

The 51 protein-coding genes in the chloroplast genome of *Chloranthus* were analyzed as a whole. By constructing high-expression and low-expression gene pools, the RSCU values for these two gene pools were calculated separately. The results showed that the optimal codon number of chloroplast genomes in *Chloranthus* ranged from 12 to 14 (Figure 8). Among them, *C. angustifolius* and *C. fortunei* had the highest number of optimal codons, while *C. henryi*, *C. nervosus*, and *C. spicatus* had the lowest number of optimal codons. The shared optimal codons in the chloroplast genomes of the *Chloranthus* species are GCU, UGU, UUA, UUG, AAA, CCU, UCU, ACU, GUA, and GUU (Figure 8). Moreover *C. henryi* had one unique optimal codon of CCA, which encoded proline. Among the shared optimal codons, only GUA encoding valine had an ∆RSCU greater than 0.5 in all of the species (Table S4). Excluding UUG, there was a preference for optimal codons ending with A/U bases in *Chloranthus*.

## 4. Discussion

### 4.1. The Chloroplast Characters and Phylogenetic Relationships Among Chloranthus

In this study, we newly obtained a chloroplast genome of *C. angustifolius*. Combined with the six published chloroplast genomes of this genus, we found that the chloroplast genome sizes of the seven species ranged between 157,063bp (*C. fortunei*) and 158,758 bp (*C. spicatus*), with a variation of 1695 bp. Additionally, 131 genes were annotated in the chloroplast genomes of all seven species, comprising 86 CDSs, 37 tRNAs, and 8 rRNAs, with 39.2–39.3% GC contents. These results implied that the chloroplast genome sequences and GC contents within the genus *Chloranthus* are conserved. The high conservatism of plastome has also been observed in other angiosperm genera [48,49].

The phylogenetic tree showed that *C. angustifolius* and *C. fortunei* were clustered together with the highest supporting values (Figure 2), which is completely consistent with the results of Yao et al. [29]. Also, based on chloroplast genomes, the division of *Chloranthus* species into two major clades is well supported (Figure 2), and these results are similar with the phylogenetic results of Kong et al. [20] and Zhang et al. [23].

### 4.2. Natural Selection Plays a Key Role in the Codons Usage of Chloranthus

The preference for synonymous codon usage is shaped by a combination of factors, including natural selection and base mutation. According to the neutral theory of molecular evolution, the impact of natural selection and base mutations on the third base of codons in an organism’s genome is mostly neutral or nearly neutral [50]. In this study, our results showed that the proportion of A/T bases in the chloroplast genomes was higher than that of G/C (Table 1; Figure 3 and Figure 7), indicating a preference for using A/U bases in codons. Previous studies have shown that the GC content at the third position of codons (GC3) can serve as an important indicator for assessing codon usage preference [51]. Furthermore, the overall GC content of codons and the GC content at each of the three codon positions in *Chloranthus* species were all less than 50%, and they exhibited a trend of GC1 > GC2 > GC3 (Table 1), which indicated an uneven distribution of codons with a preference for A/U termination [51]. The results were consistent with patterns found in most angiosperms [52]. The analysis of the ENC values in seven species showed that all of them were greater than 45 (Table 1), suggesting a relatively weak codon usage bias in the genus *Chloranthus* [41,53,54]. Additionally, there was no significant correlation between the ENC values and GC1 and GC3 (Table 2 and Table S3), which was mainly influenced by the base composition at the second position (GC2) and the length of the chloroplast gene sequence. Considering the differential usage among the three base positions, it is inferred that the codon preference characteristics of the chloroplast genomes of these seven *Chloranthus* species are more influenced by natural selection pressure than by mutation (Figure 4, Figure 5 and Figure 6) [42,43,55,56]. A previous study proposed that codons usage bias in *Delphinium grandiflorum* was shaped by natural selection [57]. Therefore, the codon bias in plants can be affected by various factors, which may differ among species. The codon usage results of *Chloranthus* species here are consistent with the other angiosperms, such as *Nymphaea* [58], Theaceae [59], Asteraceae [60], and Solanaceae [61]. The above results collectively indicate that the codon usage frequency in the chloroplast genome of the *Chloranthus* genus is relatively consistent with that of most dicotyledonous plants, suggesting an absence of significant specific changes during its long-term evolution, which may contribute to its relatively weak codon usage bias.

### 4.3. Optimal Codons Offers New Insights for Chloroplast Genetic Engineering in Chloranthus

Codon usage frequency is commonly employed to compare the codon selection preferences among different species, serving as a valuable reference for optimizing the heterologous expression of target genes [62,63]. Based on the RSCU values (RSCU > 1 and ∆RSCU ≥ 0.8) of codons in the chloroplast genomes of seven *Chloranthus* species, 10 high-frequency codons (GCU, UGU, UUA, UUG, AAA, CCU, UCU, ACU, GUA, and GUU) were identified for the shared optimal codons, with 9 ending in A/U (Figure 8). The result of a preference for codons ending in A or U is consistent with more eudicot plants [64], such as primitive angiosperm groups Magnoliaceae [65] and *Nymphaea* [58]. Furthermore, the optimal codons in plant chloroplast genomes can significantly enhance the accuracy and efficiency of amino acid translation, thereby promoting gene expression. Through our analysis of codon preference and determination of optimal codons in the *Chloranthus* chloroplast genome, this study provides a valuable reference for optimizing exogenous genes to improve their expression efficiency within the *Chloranthus* chloroplast.

## 5. Conclusions

In this study, we newly sequenced the chloroplast genome of *C. angustifolius*, which was found to be sister to *C. fortunei* with the highest support values. Our analysis of codon usage revealed a tendency for *Chloranthus* species to prefer codons ending in A or U within their chloroplast genomes, and ten high-frequency codons were identified within *Chloranthus* species. These findings provide a critical reference for optimizing codon usage and enhancing the expression efficiency of exogenous genes in future chloroplast engineering efforts within this genus. Additionally, the neutrality plot, PR2 bias plot, and ENC-plot collectively indicated that natural selection is the predominant force shaping codon usage patterns. This study offers a preliminary yet comprehensive examination of codon usage patterns and influencing factors in *Chloranthus* species. However, the relatively small sample size of seven *Chloranthus* species may not fully capture the diversity within the genus and the analysis was limited to chloroplast genomes, and further studies incorporating nuclear and mitochondrial genomes could provide a more comprehensive understanding of codon usage bias in *Chloranthus*.

## Figures and Tables

**Table 1:** Basic parameters of codon usage bias of chloroplast genome in *Chloranthus*.

Species	Genbank No.	N.	GC1	GC2	GC3	GCall	ENC	GC3_s_
*C. angustifolius*	MW581013	20 728	0.475	0.403	0.301	0.393	46.26	0.302
*C. nervosus*	NC_082096	20 728	0.475	0.403	0.301	0.393	46.32	0.302
*C. spicatus*	NC_009598	20 728	0.475	0.403	0.301	0.393	46.32	0.302
*C. japonicus*	NC_026565	20 758	0.475	0.403	0.302	0.393	46.27	0.302
*C. erectus*	NC_039627	20 719	0.473	0.403	0.300	0.392	46.47	0.301
*C. henryi*	MK922064	20 718	0.473	0.403	0.299	0.392	46.23	0.299
*C. fortunei*	NC_065306	20 721	0.474	0.403	0.301	0.393	46.29	0.302

**Table 2:** Correlation analysis of codon parameters in *Chloranthus* species.

Species	Variation	Codon Parameters				
		GC1	GC2	GC3	GCall	ENC
*C. angustifolius*	GC2	0.245				
	GC3	0.179	−0.065			
	GCall	0.808 **	0.675 **	0.408 **		
	ENC	0.218	−0.371 **	0.249	0.014	
	Codon No.	−0.103	−0.274	0.274	−0.122	0.313 *
*C. nervosus*	GC2	0.241				
	GC3	0.161	−0.046			
	GCall	0.801 **	0.677 **	0.410 **		
	ENC	0.224	−0.380 **	0.184	−0.007	
	Codon No.	−0.102	−0.274	0.272	−0.121	0.280 *
*C. spicatus*	GC2	0.241				
	GC3	0.161	−0.046			
	GCall	0.801 **	0.677 **	0.410 **		
	ENC	0.224	−0.380 **	0.184	−0.007	
	Codon No.	−0.102	−0.274	0.272	−0.121	0.280 *
*C. japonicus*	GC2	0.242				
	GC3	0.160	−0.067			
	GCall	0.806 **	0.676 **	0.393 **		
	ENC	0.19	−0.389 **	0.239	−0.016	
	Codon No.	−0.098	−0.274	0.277 *	−0.119	0.300 *
*C. erectus*	GC2	0.259				
	GC3	0.179	−0.036			
	GCall	0.807 **	0.687 **	0.419 **		
	ENC	0.192	−0.371 **	0.233	−0.008	
	Codon No.	−0.088	−0.277 *	0.290 *	−0.108	0.276 *
*C. henryi*	GC2	0.214				
	GC3	0.218	−0.036			
	GCall	0.802 **	0.687 **	0.419 **		
	ENC	0.212	−0.371 **	0.233	−0.008	
	Codon No.	−0.082	−0.277 *	0.290 *	−0.108	0.276 *
*C. fortunei*	GC2	0.231				
	GC3	0.163	−0.060			
	GCall	0.802 **	0.675 **	0.397 **		
	ENC	0.243	−0.360 **	0.214	0.023	
	Codon No.	−0.095	−0.277 *	0.268	−0.123	0.289 *

** denotes an extremely significant correlation (*p* < 0.01), * denotes a statistically significant correlation (*p* < 0.05).

## Data Availability

The chloroplast genome sequence supporting this study has been uploaded to GenBank (National Center for Biotechnology Information) with the accession number MW581013. The associated BioProject, SRA, and Bio-Sample numbers are PRJNA692276, SRR13447375, and SAMN17319709, respectively. Additional materials supporting the results of this article are included in the Supplementary Files.

## Supplementary Materials

The following supporting information can be downloaded at: https://www.mdpi.com/article/10.3390/genes16020186/s1, Table S1: List of gene contents in *C. angustifolius*; Table S2: The CDS numbers in the chloroplast genome of *Chloranthus* species; Table S3: The *p*-value of codon parameters in *Chloranthus* species. Table S4: Optimal codons in chloroplast genomes of seven *Chloranthus* species.
