# Supplementary material for: Analysis of the Codon Usage Bias Pattern in the Chloroplast Genomes of Chloranthus Species (Chloranthaceae)
Source: Genes (Basel). 2025 Feb 2;16(2):186. doi: 10.3390/genes16020186 (PMC11855406; doi:10.3390/genes16020186)
Supplement: Supplementary file 1 [file genes-16-00186-s001.zip › genes-3433189-supplementary.pdf]

Supplementary Information

Table S1. List of gene contents in *Chloranthus angustifolius*

Table S2. The CDS numbers in the chloroplast genome of *Chloranthus* species.

Table S3. The *P*-value of codon parameters in *Chloranthus* species.

Table S4. Optimal codons in chloroplast genome of seven *Chloranthus species*.

Table S1. List of gene contents in *Chloranthus angustifolius*

Category	Groups of Genes	Names of Genes
Self-replication	Ribosomal RNA	*rrn4.5**, *rrn5**, *rrn16**, *rrn23**
	Transfer RNA	*trnA-UGC**,†, *trnC-GCA*, *trnD-GUC*, *trnE-UUC*, *trnF-GAA*, *trnM-CAU**, *trnG-GCC*, *trnH-GUG*, *trnI-GAU**,†, *trnK-UUU*†, *trnL-CAA**, *trnL-UAA*†, *trnL-UAG*, *trnN-GUU**, *trnP-UGG*, *trnQ-UUG*, *trnR-ACG**, *trnR-UCU*, *trnS-GCU*, *trnS-GGA*, *trnS-UGA*, *trnT-GGU*, *trnT-UGU*, *trnV-GAC**, *trnV-UAC*†, *trnW-CCA*, *trnY-GUA*
	Small subunit of ribosome	*rps2*, *rps3*, *rps4*, *rps7**, *rps8*, *rps11*, *rps12**,†, *rps14*, *rps15*, *rps16*, *rps18*, *rps19*
	Large subunit of ribosome	*rpl2**,†, *rpl14*, *rpl16*†, *rpl20*, *rpl22*, *rpl23**, *rpl32*, *rpl33*, *rpl36*
	RNA polymerase subunit	*rpoA*, *rpoB*, *rpoC1*†, *rpoC2*
Photosynthesis	ATP synthase	*atpA*, *atpB*, *atpE*, *atpF*†, *atpH*, *atpI*
	NADH dehydrogenase	*ndhA*†, *ndhB**,†, *ndhC*, *ndhD*, *ndhE*, *ndhF*, *ndhG*, *ndhH*, *ndhI*, *ndhJ*, *ndhK*
	Cytochrome b/f complex	*petA*, *petB*†, *petD*†, *petG*, *petL*, *petN*
	PhotosystemI	*psaA*, *psaB*, *psaC*, *psaI*, *psaJ*
	PhotosystemII	*psbA*, *psbB*, *psbC*, *psbD*, *psbE*, *psbF*, *psbH*, *psbI*, *psbJ*, *psbK*, *psbL*, *psbM*, *psbT*, *psbZ*
	Rubisco large subunit	*rbcL*
Others	Translation initiation	*infA*
	Proteolysis	*clpP*†
	Cytochrome *c* biogenesis protein	*ccsA*
	Acetyl-CoA carboxylase	*accD*
	Chloroplast envelope membrane protein	*cemA*
	maturase	*matK*
	Hypothetical reading frame	*ycf1**, *ycf2**, *ycf3*†, *ycf4*

*, duplicated gene in IR; †, gene including one or two introns

Table S2. The CDS numbers in the chloroplast genome of *Chloranthus* species.

Species	CDS before filtering	CDS after filtering	CDS list
C. angustifolius	86	51	*accD, atpA, atpB, atpE, atpF, atpI, ccsA, cemA, clpP, matK, ndhA, ndhB, ndhC, ndhE, ndhF, ndhG, ndhH, ndhI, ndhJ, ndhK, petA, petB, petD, psaA, psaB, psbA, psbB, psbC, psbD, rbcL, rpl14, rpl16, rpl20, rpl22, rpoA, rpoB, rpoC1, rpoC2, rps11, rps12, rps14, rps18, rps2, rps3, rps4, rps7, rps8, ycf1, ycf2, ycf3, ycf4*
C. nervosus	86	51	
C. spicatus	86	51	
C. japonicus	86	51	
C. erectus	86	51	
C. henryi	86	51	
C. fortunei	86	51	

Table S3. The *P*-value of codon parameters in *Chloranthus* species.

**Species**	**Variation**	**Codon parameters**				
		GC1	GC2	GC3	GCall	ENC
	GC2	0.0826				
	GC3	0.209	0.65			
C. angustifolius	GCall	7.95e-13	5.69e-08	0.00298		
	ENC	0.125	0.00736	0.0781	0.921	
	Codon No.	0.47	0.0515	0.0519	0.394	0.0254
	GC2	0.0886				
	GC3	0.26	0.747			
C. nervosus	GCall	1.66e-12	4.92e-08	0.00278		
	ENC	0.114	0.00592	0.196	0.96	
	Codon No.	0.475	0.0518	0.0538	0.399	0.0466
	GC2	0.0886				
	GC3	0.26	0.747			
C. spicatus	GCall	1.66e-12	4.92e-08	0.00278		
	ENC	0.114	0.00592	0.196	0.96	
	Codon No.	0.475	0.0518	0.0538	0.399	0.0466
	GC2	0.0869				
	GC3	0.262	0.639			
C. japonicus	GCall	9.62e-13	5.26e-08	0.00438		
	ENC	0.181	0.00477	0.0906	0.911	
	Codon No.	0.495	0.052	0.0494	0.405	0.0325
	GC2	0.0665				
	GC3	0.208	0.803			
C. erectus	GCall	8.43e-13	2.65e-08	0.00223		
	ENC	0.178	0.00734	0.101	0.957	
	Codon No.	0.539	0.0488	0.0388	0.449	0.0496
	GC2	0.131				
	GC3	0.124	0.776			
C. henryi	GCall	1.57e-12	1.09e-07	0.00115		
	ENC	0.135	0.012	0.145	0.957	
	Codon No.	0.568	0.0496	0.0291	0.485	0.0464
	GC2	0.102				
	GC3	0.255	0.673			
C. fortunei	GCall	1.53e-12	5.71e-08	0.00396		
	ENC	0.0854	0.00939	0.131	0.872	
	Codon No.	0.509	0.0493	0.0569	0.391	0.0395

Table S4. Optimal codons in chloroplast genome of seven *Chloranthus species*.

Species	Amino Acid	Codon	High expression gene		Low expression gene		ΔRSCU
			RSCU	No.	RSCU	No.	
C. angustifolius	Ala	GCA*	1.29	22	1.19	34	0.10
		GCU***	2.24	38	1.72	49	0.52
	Cys	UGU*	1.71	6	1.48	31	0.23
	Gln	CAA*	1.55	17	1.43	98	0.12
	Glu	GAA*	1.41	29	1.29	130	0.12
	Ile	AUU*	1.48	36	1.36	135	0.12
	Leu	UUA**	1.75	23	1.31	84	0.44
		UUG**	1.82	24	1.45	93	0.37
	Lys	AAA*	1.54	27	1.30	125	0.24
	Pro	CCU**	1.57	20	1.14	40	0.43
	Ser	UCU**	1.93	19	1.61	95	0.32
	Thr	ACU**	1.58	19	1.26	49	0.32
	Val	GUA***	2.00	24	1.24	43	0.76
		GUU*	1.50	18	1.32	46	0.18
C. fortunei	Ala	GCA*	1.29	22	1.11	31	0.18
		GCU**	2.24	38	1.82	51	0.42
	Cys	UGU**	1.75	7	1.44	28	0.31
	Gln	CAA*	1.55	17	1.41	95	0.14
	Glu	GAA*	1.40	28	1.27	129	0.13
	Ile	AUU*	1.46	36	1.35	125	0.11
	Leu	UUA***	1.75	23	1.11	64	0.64
		UUG**	1.82	24	1.47	85	0.35
	Lys	AAA*	1.53	26	1.29	120	0.24
	Pro	CCU**	1.57	20	1.19	41	0.38
	Ser	UCU*	1.90	19	1.69	90	0.21
	Thr	ACU**	1.58	19	1.19	42	0.39
	Val	GUA***	1.96	23	1.26	43	0.70
		GUU*	1.53	18	1.35	46	0.18
C. henryi	Ala	GCU***	2.28	37	1.65	43	0.63
	Cys	UGU***	2.00	8	1.46	30	0.54
	Ile	AUU*	1.64	46	1.38	133	0.26
	Leu	UUA***	1.82	31	1.26	82	0.56
		UUG*	1.65	28	1.46	95	0.19
	Lys	AAA*	1.54	20	1.30	125	0.24
	Pro	CCA*	1.50	18	1.22	42	0.28
		CCU**	1.58	19	1.19	41	0.39
	Ser	UCU*	1.68	19	1.59	93	0.09
	Thr	ACU**	1.73	22	1.38	52	0.35
	Val	GUA***	2.00	27	1.24	47	0.76

		GUU*	1.56	21	1.42	54	0.14
C. japonicus	Ala	GCA*	1.24	21	1.15	33	0.09
		GCU***	2.24	38	1.70	49	0.54
	Cys	UGU*	1.75	7	1.48	31	0.27
	Gln	CAA*	1.55	17	1.43	98	0.12
	Glu	GAA*	1.40	28	1.27	129	0.13
	Leu	UUA**	1.75	23	1.29	83	0.46
		UUG**	1.82	24	1.47	94	0.35
	Lys	AAA*	1.47	25	1.30	126	0.17
	Pro	CCU**	1.57	20	1.16	40	0.41
	Ser	UCU*	1.90	19	1.63	96	0.27
	Thr	ACU**	1.58	19	1.27	50	0.31
	Val	GUA***	1.92	23	1.23	43	0.69
		GUU*	1.58	19	1.31	46	0.27
C. nervosus	Ala	GCU***	2.27	38	1.71	45	0.56
	Cys	UGU**	1.75	7	1.42	27	0.33
	Gln	CAA*	1.55	17	1.39	91	0.16
	Glu	GAA*	1.41	29	1.29	131	0.12
	Leu	UUA***	1.75	23	1.07	63	0.68
		UUG**	1.82	24	1.50	88	0.32
	Lys	AAA*	1.53	26	1.29	121	0.24
	Pro	CCU**	1.57	20	1.13	40	0.44
	Ser	UCU*	1.93	19	1.65	90	0.28
	Thr	ACU**	1.55	19	1.20	40	0.35
	Val	GUA***	1.92	23	1.23	44	0.69
		GUU*	1.58	19	1.45	52	0.13
C. spicatus	Ala	GCU***	2.27	38	1.71	45	0.56
	Cys	UGU**	1.75	7	1.42	27	0.33
	Gln	CAA*	1.55	17	1.39	91	0.16
	Glu	GAA*	1.41	29	1.29	131	0.12
	Leu	UUA***	1.75	23	1.07	63	0.68
		UUG**	1.82	24	1.50	88	0.32
	Lys	AAA*	1.53	26	1.29	121	0.24
	Pro	CCU**	1.57	20	1.13	40	0.44
	Ser	UCU*	1.93	19	1.65	90	0.28
	Thr	ACU**	1.55	19	1.20	40	0.35
	Val	GUA***	1.92	23	1.23	44	0.69
		GUU*	1.58	19	1.45	52	0.13
C. erectus	Ala	GCU***	2.24	38	1.68	44	0.56
	Cys	UGU**	1.75	7	1.38	29	0.37
	Gln	CAA*	1.62	17	1.37	92	0.25
	Glu	GAA*	1.41	29	1.31	133	0.10
	Ile	AUU*	1.46	35	1.33	123	0.13

Leu	UUA***	1.75	23	1.03	62	0.72
	UUG**	1.82	24	1.51	91	0.31
Lys	AAA*	1.49	26	1.30	125	0.19
Pro	CCU**	1.65	21	1.18	41	0.47
Ser	UCU**	1.90	19	1.58	87	0.32
Thr	ACU*	1.58	19	1.32	46	0.26
Val	GUA***	2.04	25	1.25	42	0.79
	GUU*	1.55	19	1.34	45	0.21

* $0.08 \leq \Delta RSCU < 0.30$, ** $0.30 \leq \Delta RSCU < 0.50$, *** $0.50 \leq \Delta RSCU$
